# Remnants of horizontal transfers of *Wolbachia* genes in a *Wolbachia*-free woodwasp

**DOI:** 10.1186/s12862-022-01995-x

**Published:** 2022-03-26

**Authors:** Joséphine Queffelec, Alisa Postma, Jeremy D. Allison, Bernard Slippers

**Affiliations:** 1 Forestry and Agricultural Biotechnology Institute, University of Pretoria, Lunnon Road, Pretoria, 0002 South Africa; 2 Department of Biochemistry, Genetics and Microbiology, University of Pretoria, Pretoria, South Africa; 3 Great Lakes Forestry Center, Natural Resources Canada, Canadian Forest Service, Sault St Marie, Canada; 4 Department of Zoology and Entomology, University of Pretoria, Pretoria, South Africa

**Keywords:** Horizontal gene transfer, *Wolbachia*, Siricidae, Hymenoptera

## Abstract

**Background:**

*Wolbachia* is a bacterial endosymbiont of many arthropod and nematode species. Due to its capacity to alter host biology, *Wolbachia* plays an important role in arthropod and nematode ecology and evolution. *Sirex noctilio* is a woodwasp causing economic loss in pine plantations of the Southern Hemisphere. An investigation into the genome of this wasp revealed the presence of *Wolbachia* sequences. Due to the potential impact of *Wolbachia* on the populations of this wasp, as well as its potential use as a biological control agent against invasive insects, this discovery warranted investigation.

**Results:**

In this study we first investigated the presence of *Wolbachia* in *S. noctilio* and demonstrated that South African populations of the wasp are unlikely to be infected. We then screened the full genome of *S. noctilio* and found 12 *Wolbachia* pseudogenes. Most of these genes constitute building blocks of various transposable elements originating from the *Wolbachia* genome. Finally, we demonstrate that these genes are distributed in all South African populations of the wasp.

**Conclusions:**

Our results provide evidence that *S. noctilio* might be compatible with a *Wolbachia* infection and that the bacteria could potentially be used in the future to regulate invasive populations of the wasp. Understanding the mechanisms that led to a loss of *Wolbachia* infection in *S. noctilio* could indicate which host species or host population should be sampled to find a *Wolbachia* strain that could be used as a biological control against *S. noctilio*.

**Supplementary Information:**

The online version contains supplementary material available at 10.1186/s12862-022-01995-x.

## Background

*Wolbachia* is a symbiont of many arthropod and filarial nematode species. This alphaproteobacteria in the family Anaplasmataceae is estimated to infect over 50% of terrestrial arthropods [1–3]. Due to its ubiquity and its effects on host reproduction and physiology, *Wolbachia* can have significant impacts on arthropod and nematode evolution [4].

*Wolbachia* uses a variety of mechanisms to modify the reproductive biology of its host and to enhance its chances of maternal transmission [5]. These mechanisms include male killing [6], feminization of genetic males [7], parthenogenesis induction [8] and cytoplasmic incompatibility that prevents embryonic development in crosses between a *Wolbachia*-positive male and a female that does not carry *Wolbachia*, or carries a different *Wolbachia* strain [9]. A *Wolbachia* infection can also provide advantages including resistance against viruses [10] and facilitating host iron metabolism [11].

A common characteristic of the *Wolbachia*-host interaction is Horizontal Gene Transfers (HGTs) from the *Wolbachia* genome to the host genome [12]. Thus far, over 20 species of nematodes, insects and isopods have been shown to carry *Wolbachia* genes in their genomes [13–19]. The transferred genetic elements vary in size from single genes to full genomes [15]. It is hypothesized that those HGTs were facilitated by the fact that the bacterial symbiont resides in the germline of the female host [12].

The genetic elements transferred from *Wolbachia* to their hosts sometimes include genes belonging to bacteriophages such as the *Wolbachia*-specific WO bacteriophages [19]. These bacteriophages play a crucial part in the *Wolbachia*-arthropod relationship [20]. It has been hypothesized that the phages can increase *Wolbachia* virulence and may be responsible for part of the molecular processes behind feminization of genetic males [21] and cytoplasmic incompatibility [22]. In order to be integrated into bacterial genomes, these viruses use specialized proteins that could also be responsible for the horizontal gene transfer of WO phage and *Wolbachia* genes into the hosts’ genomes [23].

The woodwasp, *Sirex noctilio* Fabricus (Hymenoptera: Siricidae) originates from Europe, Eurasia and Northern Africa [24] and has been introduced in many countries over the last century [25]. Today, it is a very successful invader and a pest in many of the Southern Hemisphere pine forests [26]. Research into control strategies of the wasp has included the sequencing of its genome (Postma et al., unpublished). Analysis of the newly sequenced genome led to the identification of gene sequences apparently originating from *Wolbachia*. Because of the potential to use *Wolbachia* as a biological control agent against insect populations [27], this finding warranted further investigation.

In this study we investigated the presence of *Wolbachia* in South African populations of *S. noctilio*. We also investigated whether the *Wolbachia* genes observed in the genome of *S. noctilio* could have been horizontally transferred into the *S. noctilio* genome. We screened the entire *S. noctilio* genome to locate potentially horizontally transferred genes from *Wolbachia*. Finally, we screened individuals from different South African populations of the woodwasp using specifically designed PCR primers for the presence of the identified genes.

## Results

### Presence of *Wolbachia* in *S. noctilio*

To test for the presence of *Wolbachia* in *S. noctilio*, 14 primers targeting three *Wolbachia* genes were used (Tables 1 and 2), along with a series of protocols that used three DNA extraction methods, two different *Taq* polymerases and a total of four cycling protocols with different annealing temperatures (Additional file 11: Table S1).

**Table 1:** Primers used

Primer	Target gene	Primer sequence (5′–3′)	References
Wspecf	16S	CATACCTATTCGAAGGGATAG	Werren and Windsor 2000
Wspecr	16S	AGCTTCGAGTGAAACCAATTC	Werren and Windsor 2000
pA (27 F)	16S	AGAGTTTGATCMTGGCTCAG	Edwards et al. 1989
EHR 16SR	16S	GTAATCGTGGATCATCATGC	Parola et al. 2000
EHR 16SD	16S	GGTACCYACAGAAGAAGTCC	Parola et al. 2000
pH (1492 R)	16S	TACGGYTACCTTGTTACGACTT	Reysenbach et al. 1992
16S 567F	16S	ATYATTGGGCGTAAAGGG	This study
16S 712F	16S	TATTAGGAGGAACACCRGT	This study
16S 712R	16S	ACYGGTGTTCCTCCTAATA	This study
16S 1401R	16S	AGTGTGTACAAGACCCGAG	This study
*Wsp* 81 F	*wsp*	TGGTCCAATAAGTGATGAAGAAAC	Braig et al. 1998
*Wsp* 691 R	*wsp*	AAAAATTAAACGCTACTCCA	Braig et al. 1998
*ftsZ*f1	*FtsZ*	GTTGTCGCAAATACCGATGC	Werren et al. 1995
*ftsZ*r1	*FtsZ*	CTTAAGTAAGCTGGTATATC	Werren et al. 1995
SnW1f	ORF4	TACCGCCAAAGTGTTCATCA	This study
SnW1r	ORF4	TGCCATCTGGTGAAATTGAA	This study
SnW2f	ORF5	TCCATAAGTGGGCTCTCACC	This study
SnW2r	ORF5	AGAGCCGAACGCTTATATGG	This study
SnW3f	ORF8	CACACCTTCTGGAATGCTGA	This study
SnW3r	ORF8	AAAGTTGCGCTACCTGATGG	This study

**Table 2:** Primer combinations, annealing temperatures and amplicon sizes

Target species	Forward primer	Reverse primer	Product size (bp)	Tm (°C)
*Wolbachia*	Wspecf	Wspecr	438	57
Anaplasmataceae	pA (27F)	EHR 16SR	790	59
Anaplasmataceae	EHR 16SD	pH (1492R)	1030	60
Anaplasmataceae	16S 567F	16S 712R	145	56
Anaplasmataceae	16S 567F	16S 1401R	834	56
Anaplasmataceae	16S 712F	16S 1401R	689	57
Bacteria	pA (27F)	pH (1492R)	1465	58
*Wolbachia*	Wsp 81 F	Wsp 691 R	610	55
*Wolbachia*	*ftsZ*f1	*ftsZ*r1	1043–1055	55
*S. noctilio*	SnW1f	SnW1r	420	52
*S. noctilio*	SnW2f	SnW2r	210	56
*S. noctilio*	SnW3f	SnW3r	200	55

The general bacterial primers pA (27F) and pH (1492R) consistently produced multiple amplicons across all tested protocols. This prevented the determination of the nucleotide sequence of the amplicons and the identification of the amplified products through sequencing analysis without fragment separation or cloning.

The Anaplasmataceae-specific primers, EHR 16SD and pH (1492R) and 16S 712F and 16S 1401R amplified two bands when tested with the positive control. These combinations of primers were not used further. Primers pA (27F) and EHR 16SR amplified the right target sequence in the positive control (i.e. *Wolbachia* 16S rRNA gene). However, when tested on *S. noctilio*, the amplicons obtained had high sequence similarity with Hymenoptera sequences. Primers 16S 567F and 16S 712R and 16S 567F and 16S 1401R amplified the right target sequence in the positive control (i.e., *Wolbachia* 16S rRNA gene). Amplicons from *S. noctilio* samples grouped with 16S rRNA gene sequences of bacterial species other than *Wolbachia*.

Primers Wspecf and Wspecr, *Wsp* 81 F and *Wsp* 691 R and *ftsZ*f1 and *ftsZ*r1, respectively, amplified the 16S rRNA, *Wsp* and *FtsZ* genes of *Wolbachia* in the positive controls, but did not amplify anything from *S. noctilio* samples.

### Horizontal gene transfer from *Wolbachia* to *S. noctilio*

The first genome-wide searching method used to localize *Wolbachia* gene sequences used 14 *Wolbachia* genomes for a BLASTn analysis against the *S. noctilio* genome. This search found open reading frames (ORFS) similar to *Wolbachia* gene sequences in scaffolds 13, 62, 126 and 1255 of the annotated genome of *S. noctilio*. The second method, that used taxonomic classification of genomic DNA reads from *S. noctilio*, found ORFs similar to *Wolbachia* gene sequences in seven scaffolds (scaffolds 13, 15, 62, 79, 106, 126 and 1224). The whole genome alignment using MUMmer identified scaffold 1 as potentially carrying *Wolbachia gene* sequences.

Using the scaffolds previously identified for a BLASTx against the protein database of NCBI showed that scaffolds 1, 62, 1224 and 1255 did not contain identifiable *Wolbachia* gene sequences. When restricting the reference database to *Wolbachia* protein sequences, the BLASTx analysis found similarity between a fragment of scaffold 1224 and two *Wolbachia* protein sequences. However, the percent identity (maximum value 44.38%) was lower than when the same fragment was compared to arthropod protein sequences (minimum percent identity 56.95%).

**Fig. 1:**
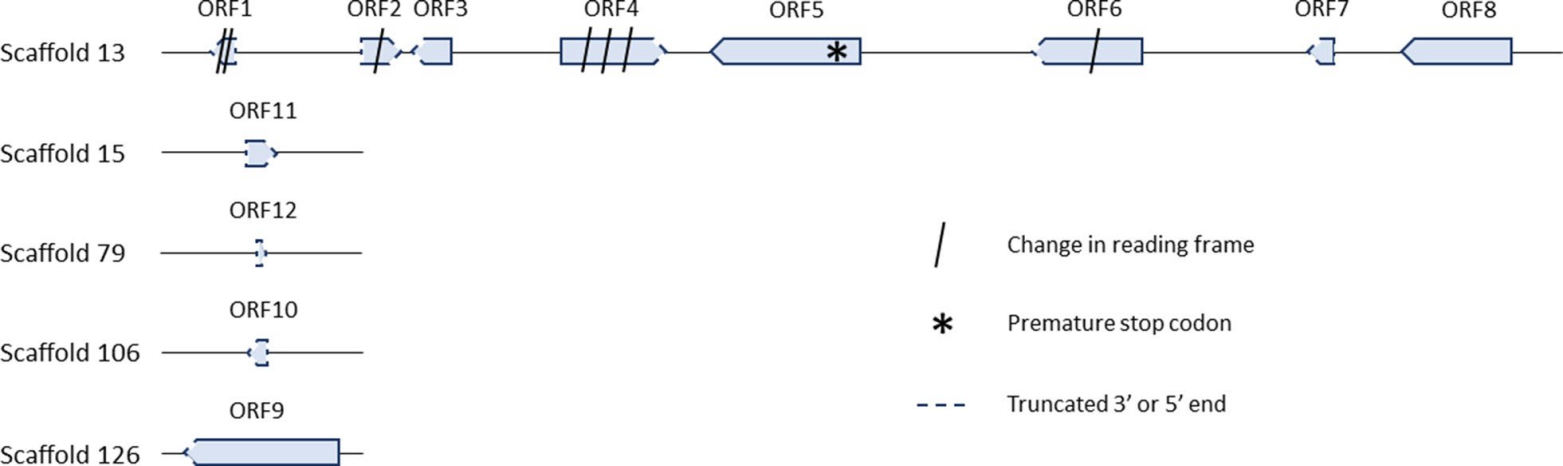
Graphic representation of the ORFs found on scaffolds 13, 15, 79, 106 and 126. The direction of the arrow indicates the direction of transcription

Across the scaffolds 13, 15, 79, 106 and 126, the BLASTx analysis found a total of 12 ORFs similar to *Wolbachia* gene sequences (Fig. 1). Eleven ORFs were either missing the 5’ or the 3’ end of the gene sequence, contained a premature stop codon or were fragmented across multiple reading frames. Only ORF8 was of the same length as the reference sequences. However, the percent identity was low (maximum percent identity 73.65%).

**Fig. 2:**
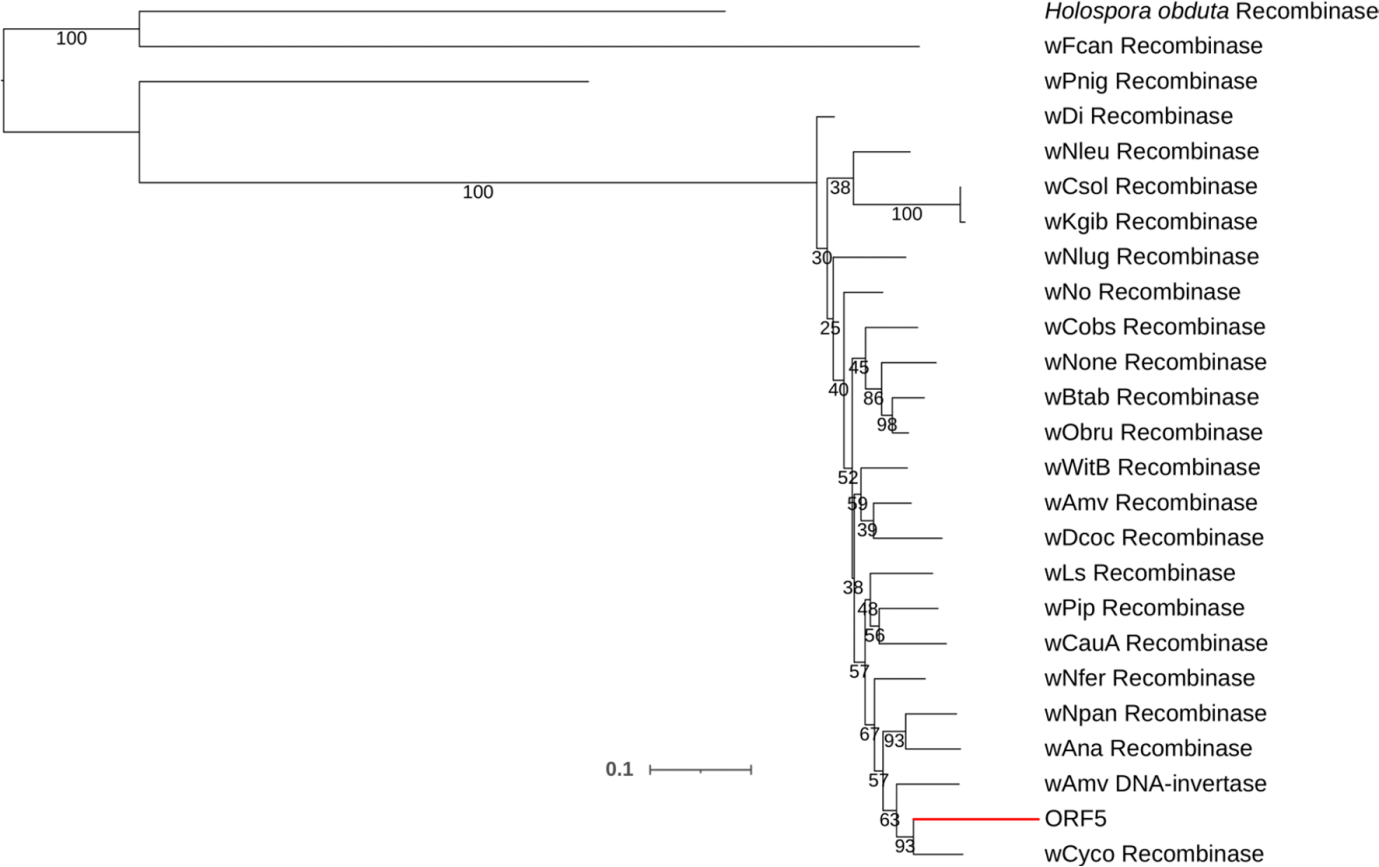
Maximum likelihood tree. It was constructed with the protein sequence of ORF5 compared to similar protein sequences of 21 *Wolbachia* strains and one protein sequence from *Holospora obducta* (Alphaproteobacteria: Holosporaceae) (out group). The branch indicated in red represents the position of ORF5 among other *Wolbachia* protein sequences. All *Wolbachia* strains are named after their hosts as follows: wAmv, *Armadillidium vulgaraei;* wAna, *Drosophila ananassae*; wBtab, *Bemisia tabaci*; wCauA, *Carposina sasakii*; wCobs, *Cardiocondyla obscurior*; wCsol, *Ceratosole solmsi*; wCyco *Cylisticus convexus*; wDcoc*, Dactylopius coccus*; wDi, *Diaphorina citri*; wFcan*, Folisomia candida*; wKgib, *Kradiba gibbosae*; wLs, *Laodelphax striatellus*; wNfer*, Nomada ferruginata*; wNleu, *Nomada leucophthalma*; wNlug, *Nilaparvata lugens*; wNo, *Drosophila simulens*; wOne, *Nasonia oneida*; wNpan, *Nomada panzeri*; wOb, *Operophtera brumata*; wPip, *Culex quinquefasciatus*; wPnig, *Pentalonia nigrinervosa*; wVitB, *Nasonia vitripennis*

**Fig. 3:**
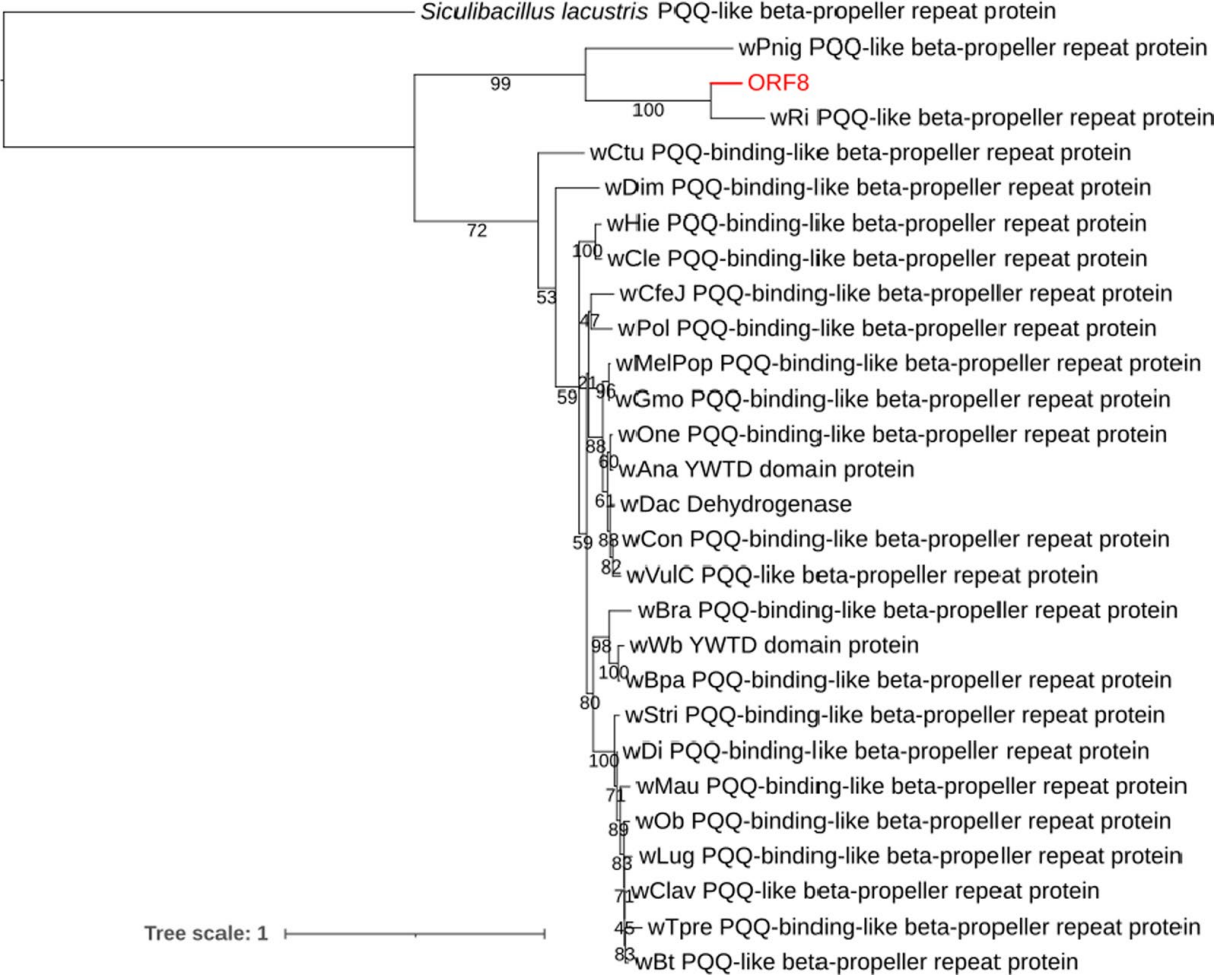
Maximum likelihood tree. It was constructed with the protein sequence of ORF8 compared to similar protein sequences of 26 *Wolbachia* strains and one protein sequence from *Siculibacillus lacustris* (Alphaproteobacteria: Ancalomicrobiaceae) (out group). The branch indicated in red represents the position of ORF8 among other *Wolbachia* protein sequences. All *Wolbachia* strains are named after their hosts as follows: wAna, *Drosophila ananassae*; wBpa, *Brugia pahangi*; wBra, *Litomosoides brasiliensis*; wBt, *Bemisia tabaci*; wCfeJ, *Ctenocephalides felis*; wClav, *Leptopilina clavipes*; wCle, *Cimex lectularius*; wCon, *Cylisticus convexus*; wCtu, *Cruorifilaria tuberocauda*; wDac, *Dactylopius coccus*; wDi, *Diaphorina citri*; wDim, *Dirofilaria immitis*; wGmo, *Glossina morsitans*; wHie, *Madathamugadia hiepei*; wLug, *Nilaparvata lugens*; wMau, *Drosophila mauritiana*; wMelPop, *Drosophila melanogaster*; wOb, *Operophtera brumata*; wOne, *Nasonia oneida*; wPnig, *Pentalonia nigronervosa*; wPol, *Atemnus politus*; wRi, *Drosophila simulans*; wStri, *Laodelphax striatellus*; wTpre, *Trichogramma pretiosum*; wVulC, *Armadillidium vulgare*; wWb, *Wuchereria bancrofti*

**Fig. 4:**
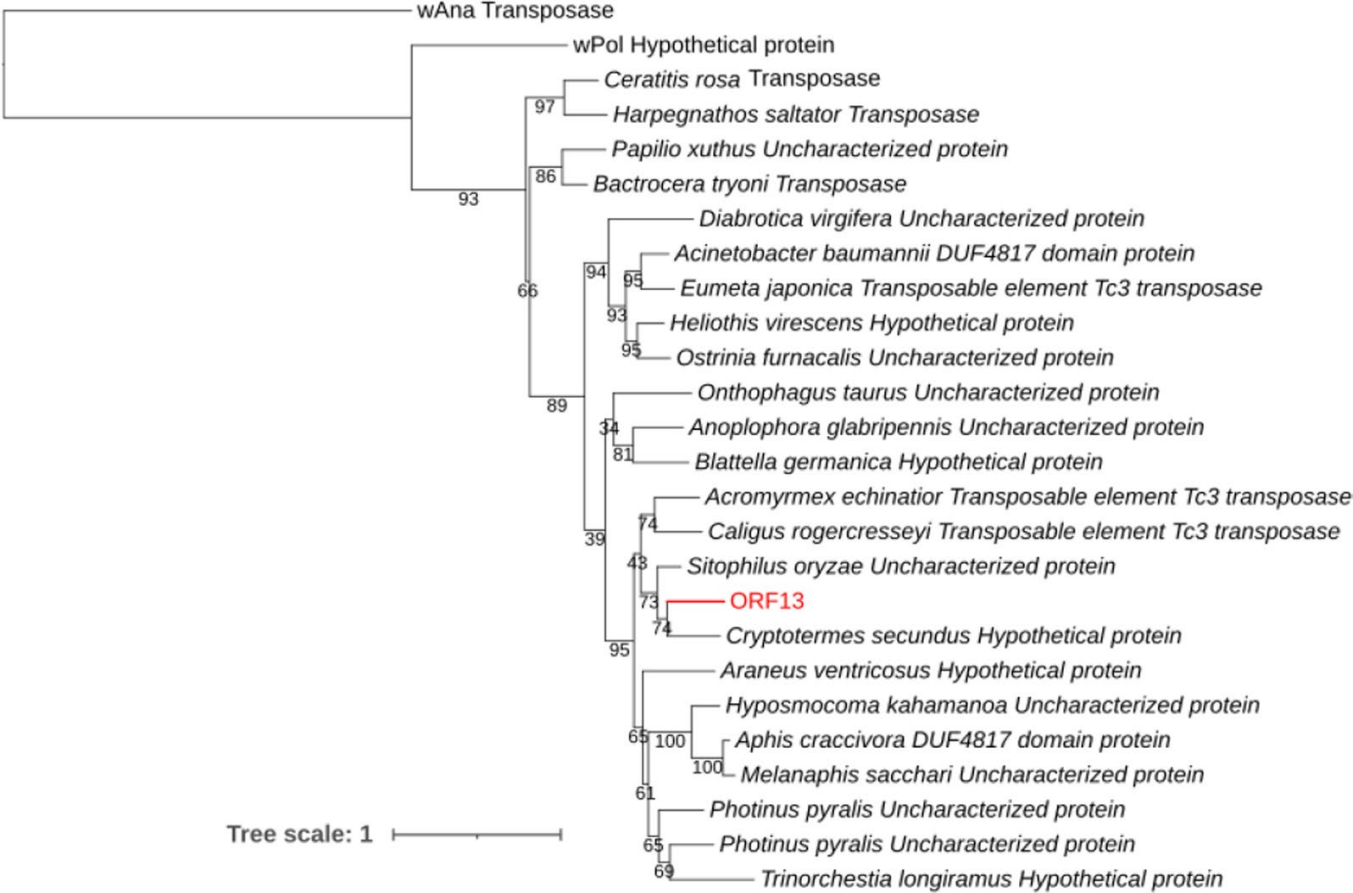
Maximum likelihood tree. It was constructed with the protein sequence of ORF13 compared to similar protein sequences from arthropod and bacterial species. The branch indicated in red represents the position of ORF13 among other sequences. The two *Wolbachia* strains are named after their hosts as follows: wAna, *Drosophila ananassae*; wPol, *Atemnus politus*

The individual gene phylogenies showed that ORF1 to ORF12 clustered with *Wolbachia* genes (Fig. 2, Fig. 3 and Additional file 1: Fig. S1, Additional file 2: Fig. S2, Additional file 3: Fig. S3, Additional file 4: Fig. S4, Additional file 5: Fig. S5, Additional file 6: Fig. S6, Additional file 7: Fig. S7, Additional file 8: Fig. S8, Additional file 9: Fig. S9, Additional file 10: Fig. S10) while ORF13 clustered with arthropod gene sequences (Fig. 4). ORF1, ORF10, ORF11 and ORF12 all shared sequence similarity with *Wolbachia* proteins containing tetratricopeptide (percent identity: 83.33%, 80%, 80% and 86.36%, respectively) and ankyrin repeats (percent identity: 83.33%, 80%, 80% and 77.27%, respectively). ORF1 and ORF10 were also similar to the phosphocholine transferase AnkX (percent identity: < 50% for both ORFs). Finally, ORF10 was also similar to a latrotoxin-related protein (percent identity: 68%). ORF2 and ORF4 showed sequence similarity with transposases of the IS4 family. ORF3 and ORF5 clustered with proteins from the recombinase family. ORF6 clustered with phage tail proteins while ORF7 showed sequence similarity with a phage related protein. ORF8 clustered with a PQQ binding-like beta propeller repeat protein and shared sequence similarity with a dehydrogenase and a YWTD domain protein (percentage identity: 75%, 41.52% and 40.22% respectively).

Finally, ORF9 shared sequence similarity with reverse transcriptases, RNA-directed DNA polymerases and Group II intron-encoded proteins (percentage identity: 95.26%, 80.18% and 73.76%, respectively).

### Ubiquity of horizontally transferred genes in *S. noctilio* in South Africa

The six primers designed in this study to amplify the horizontally transferred *Wolbachia* genes found in *S. noctilio* (i.e., SnW1f and SnW1r, SnW2f and SnW2r and SnW3f and SnW3r) all amplified the target loci. Primers SnW1f and SnW1r were arbitrarily chosen for the rest of the analysis. Out of the 500 samples collected from five South African populations, only 85 did not amplify after the first PCR, but showed amplification after dilution of the DNA samples.

## Discussion

The aim of this study was to characterise *Wolbachia* in *S. noctilio* or *Wolbachia* genes in the genome assembly of *S. noctilio*. PCR was first used to demonstrate that *S. noctilio* is unlikely to be infected with *Wolbachia*, suggesting that the genes were introgressed in the *S. noctilio* genome. Through a genome wide search and a series of local BLASTx analyses, 13 potentially horizontally transferred *Wolbachia* genes were then identified. Using individual gene phylogenies, 12 were confirmed to be *Wolbachia* genes, while one was shown to be an arthropod gene. Finally, we demonstrated that these horizontally transferred *Wolbachia* genes are present in all populations of *S. noctilio* in South Africa.

None of the PCR protocols tested in this study lead to the amplification of the *Wolbachia* genes *Wsp*, *FtsZ* or 16S rRNA. This suggests the absence of a free living *Wolbachia* in *S. noctilio* in South Africa. The protocols tested included nine different primer pairs, three DNA extraction methods, two *Taq* polymerases and a total of four cycling protocols. *Wolbachia*-specific primers are known for their high false negative rates due to a high variability in gene sequences between *Wolbachia* strains [28]. For this reason, only three *Wolbachia*-specific primer pairs were tested in this study, namely Wspecf and Wspecr, *Wsp* 81F and *Wsp* 691 R and *FtsZ*f1 *and FtsZ*r1. The remaining eight primers, including pA (27F), EHR 16SR, EHR 16SD and pH (1492R) found in the literature [29–32] and 16S 576F, 16S 712F, 16S 712R and 16S 1401R that were designed in this study, either target all bacterial species or species within the Anaplasmataceae. The broader targeted species range of these primers was tested to account for the high sequence variability among *Wolbachia* strains and might be useful for future studies on *Wolbachia* infections. When tested on DNA extracted from the *Wolbachia-*positive *A. pipithiensis*, primers pA (27F) and EHR 16SR, 16S 576F and 16S 712R and 16S 576F and 16S 1401R amplified the 16S rRNA gene from *Wolbachia*. However, when tested on DNA extracted from *S. noctilio*, the same primers amplified non-target sequences.

A total of 12 *Wolbachia* genes were found in the genome of *S. noctilio* (Fig. 1). In total, the 12 confirmed *Wolbachia* gene sequences are distributed across five different scaffolds within the genome assembly. Out of the 12 genes identified, eleven are pseudogenes as they are spread across different reading frames or contain premature stop codons (Fig. 1). These results confirm that these genes were horizontally transferred from *Wolbachia* to the *S. noctilio* genome and that these horizontal transfers are not recent (i.e., due to extensive mutation of the gene sequence). Investigating the presence of these *Wolbachia* genes in other populations of *S. noctilio* or in related species could give an indication of the time frame within which these transfers happened.

The phylogenetic analysis gave a first indication of the original function of the horizontally transferred genes in *Wolbachia*. ORF1, ORF10, ORF11 and ORF12 were similar to protein sequences containing tetratricopeptide and ankyrin repeats. This category includes the phosphocholine transferase AnkX [33]. These repeats enable protein–protein interactions in eukaryotic cells [34]. In *Wolbachia*, these genes are part of the *Wolbachia* bacteriophages WO [20], a group of temperate double-stranded DNA phages that use *Wolbachia* as a host [35]. The genes that contain ankyrin and tetratricopeptide repeats are located in the “eukaryotic association module” of the bacteriophage WO genome [20] and are involved in host biology manipulation [21, 22].

The phylogenetic analysis showed that ORF10 shares some sequence similarity with a latrotoxin related protein (Additional file 8: Fig. S8). Latrotoxins are an important component of the venom of the widow spiders in the genus *Latrodectus* [36]. However, C-terminal domain homologs of the latrotoxin gene are part of the “eukaryotic association module” of the phage WO [20]. Latrotoxin genes might have been acquired by WO bacteriophages through horizontal gene transfer and are now potentially used for eukaryotic host cell disintegration. The horizontal gene transfer of C-terminal domain latrotoxins from a *Wolbachia* strain to its host was also demonstrated in the genomes of the *Wolbachia*-positive *Halyomorpha halys* [18] and *Aedes aegypti* [20].

ORF2 and ORF4 were similar to IS4-family transposases. Insertion elements, such as the ones belonging to the IS4 family, are a type of transposable element widely distributed among bacterial genomes [37, 38]. Their capacity to move to other loci in the genome is mediated by a transposase [39].

ORF3 and ORF5 both clustered with proteins of the recombinase family. Recombinases are proteins essential for genome replication in bacteria and are also crucial components of mobile genetic elements such as integrons, plasmids, transposons and bacteriophages [40]. Recombinases can lead to the integration of new DNA sequences in the host genome through strand exchange between the mobile genetic element and the target sequence in the host genome. ORF5 also clustered with a DNA invertase, a type of recombinase protein [41].

ORF9 showed sequence similarity to group II intron reverse transcriptases/maturases and RNA-directed DNA polymerases. These proteins indicate that ORF9 might be a specific type of reverse transcriptase found in bacteria, called retrointrons [42]. These types of retroelements can integrate into a DNA strand by binding to the host DNA as retrointron RNA and by being reverse transcribed into the target DNA strand [43].

ORF8 clustered with proteins with PQQ and YWTD domains. These domains are present in β-propeller proteins, a group of homologous proteins with a characteristic central “barrel” surrounded by a varying number of twisted β-sheets that form “blades” [44]. These proteins are found in viruses, bacteria, archaea and eukaryotes and assume a wide variety of functions [45]. The fact that ORF8 also clustered with a dehydrogenase indicated that, in *Wolbachia*, ORF8 could have taken part in the oxidation of methanol or ethanol, functions sometimes executed by proteins with a PQQ domain [44].

ORF6 and ORF7 both clustered with phage related proteins. While the function of ORF7 cannot be determined, ORF6 clustered with phage tail proteins. These proteins are the building blocks of the phage tail involved in adsorption to and infection of the bacterial host [46].

While further functional studies would be necessary to determine the exact functions of the 12 *Wolbachia* protein coding genes found in *S. noctilio*, the phylogenetic analysis gave a first indication of how these horizontal gene transfers occurred. ORF2, ORF3, ORF4, ORF5 and ORF9 seem to be genes directly involved in transposition of various types of mobile genetic elements, such as retrointrons, transposons and bacteriophages. These genes have the capacity to introgress themselves into new host genomes. On the other hand, ORF1, ORF6, ORF7, ORF8, ORF10, ORF11 and ORF12 do not have this capacity. ORF1, ORF6, ORF7, ORF10, ORF11 and ORF12 seem to be part of the *Wolbachia* bacteriophage WO while ORF8 does not seem to be part of any transposable element, but part of the core *Wolbachia* genome. In scaffold 13 ORF1, ORF6, ORF7 and ORF8 were found in the flanking regions of ORF5 (Fig. 1) indicating that these genes might have hitch-hiked with ORF5 from the *Wolbachia* genome to the genome of *S. noctilio* [22, 46].

Horizontal gene transfers from *Wolbachia* to arthropod hosts putatively resulting in host genome evolution and expansion [47–49] and gene acquisition [12] events, have been observed in a number of studies. In *S. noctilio*, the fragments transferred from *Wolbachia* to the genome of the wasp are unlikely to have such impact. The fragments are relatively small, spanning a total of 8957 bp and have gone through substantial sequence variation.

Observing horizontally transferred *Wolbachia* genes in a *Wolbachia*-free insect species is interesting. These results demonstrate that the source population from which *S. noctilio* was introduced in South Africa carried *Wolbachia* at some point in its evolutionary history. This population could have lost the infection either prior to introduction in South Africa or after introduction and during the invasion process. An investigation into the presence of *Wolbachia* in native populations of *S. noctilio* would shed light onto the mechanisms that led to South African populations of *S. noctilio* to be *Wolbachia*-free.

It is possible that the source population from which *S. noctilio* was introduced into South Africa had lost *Wolbachia* before introduction*.* Werren and Windsor [32] and Bailly-Bechet et al. [50] have investigated the global equilibrium in *Wolbachia* incidence in arthropod species. They concluded that the loss of a *Wolbachia* infection is part of the *Wolbachia*-host interaction, and that arthropod species lose their *Wolbachia* infection more often than they acquire a new one. The mechanisms by which *Wolbachia* is lost still require investigation. There is evidence that once a *Wolbachia* strain is fixed into an arthropod population, the mechanisms by which it spread, such as cytoplasmic incompatibility, are relieved of their selective pressures and eventually erode [51]. Hornett et al. [52] have also shown that *Hypolimnas bolina* (Lepidoptera: Nymphalidae) evolved resistance against male-killing by a *Wolbachia* strain. Without a mechanism to efficiently spread through a population, *Wolbachia* could then slowly be removed from the host population.

It is possible that *S. noctilio* lost its *Wolbachia* infection over the course of the invasion process in South Africa or elsewhere. This phenomenon has been observed in the Argentine ant *Linepithema humile* after its introduction in Australia, Spain and France [53]. This loss could have happened through a founder effect. In South Africa, populations of *S. noctilio* were founded by a small number of individuals [54]. It is possible that none of the founding females carried *Wolbachia*. If the founding individuals carried *Wolbachia*, in such a small, introduced population, drift could have also led to a loss of infection through stochastic events. Finally, the *Wolbachia* infection could have been selected against during establishment and invasion. Environmental conditions such as temperature and nutrition affect *Wolbachia* titers in hosts, decreasing the capacity of the bacteria to get transferred from mother to offspring [55, 56]. Because the population of *S. noctilio* was introduced with a very low genetic diversity, a *Wolbachia* strain causing cytoplasmic incompatibility could have also been selected against as it would prevent cross fertilization.

The mechanisms by which *S. noctilio* lost its *Wolbachia*-infection has implications for the potential use of *Wolbachia* as a biological control agent against *S. noctilio*. If *S. noctilio* lost its *Wolbachia* infection because the *Wolbachia* strain it used to carry was no longer able to induce reproductive parasitism, closely related species of wood wasps might carry *Wolbachia* strains which may still have this ability. These strains could be good candidates for a biological control program. However, if *S. noctilio* lost *Wolbachia* because the wasp evolved a resistance mechanism against the bacteria, reintroducing *Wolbachia* in *S. noctilio* would be more challenging. Thankfully, *Wolbachia* strains have very different effects on hosts. For example, ten strains of *Wolbachia* have already been artificially introduced in *A. aegypti*, a mosquito species that rarely carries *Wolbachia* in the wild [27, 57]. Those strains have various effects on the reproductive biology, ecology and physiology of *A. aegypti*. As such, *S. noctilio* might be resistant to some *Wolbachia* strains but could be susceptible to others.

If *S. noctilio* lost its *Wolbachia* infection during invasion due to stochastic events related to the specific population dynamics of small populations, it might be possible to artificially introduce the *Wolbachia* strain from the population of origin into South Africa. Due to the distribution of pine trees in South Africa, the distribution of *S. noctilio* is patchy. This, along with the fact that *S. noctilio* is a haplodiploid species would slow down the spread of *Wolbachia* between populations [58, 59]. However, this could be remedied through multiple releases of infected individuals. Finally, if *S. noctilio* lost its *Wolbachia* infection due to unfavourable environmental conditions, *Wolbachia* strains potentially present in other pine pests in South Africa could be of interest.

## Conclusions

The presence of *Wolbachia* genes in the genome of *S. noctilio* suggests that *S. noctilio* is a potential host for *Wolbachia*. This could be determined by investigating the presence of *Wolbachia* in other populations of *S. noctilio*, either in the native range or in the introduced range. Because of its capacity to cause cytoplasmic incompatibility, *Wolbachia* has been investigated as a way to control mosquito populations [27] and might also help to control other insect pests in the future [60, 61]. As such, *Wolbachia* could offer new solutions for the regulation of *S. noctilio* in the Southern Hemisphere.

## Material and methods

### Presence of* Wolbachia* in *S. noctilio*

### Sample collection and storage

Logs of *Pinus patula* and *Pinus radiata* infected with *S. noctilio* were collected in 2016 and brought to the Biocontrol Centre of the Forestry and Agricultural Biotechnology Institute (FABI), at the University of Pretoria, South Africa. The logs were placed in emergence cages and emerging adults were collected. A total of 32 individuals were dissected in sterile conditions to sample testes from 17 males and eggs from 15 females. *Wolbachia*-positive fig wasps, *Alfonsiella pipithiensis* (Hymenoptera: Agaonidae) [62] were used as positive control. The wasps were collected in 2018 on the University of Pretoria Hatfield Campus by dissecting figs from *Ficus craterostoma* trees.

### DNA extraction

Three DNA extraction kits were tested on eggs and testes using the manufacturer’s instructions. The *prep*GEM Insect DNA extraction kit (ZyGEM Corporation Ltd, Hamilton, New Zealand) was used on 14 male samples and two female samples, the Zymo Quick DNA Fecal/Soil Microbe kit (Zymo Research, California, USA) was used on three male samples and the NucleoSpin DNA purification kit (Macherey–Nagel, Düren, Germany) was used on 13 female samples.

### PCR

*Wolbachia-*specific primers previously designed in the literature have low success rates due to *Wolbachia* gene sequences being highly variable among *Wolbachia* strains [28]. For this reason, 14 different primers targeting the *wsp*, the *FtsZ* and the 16S rRNA genes were tested (Table 1 and associated references and Table 2). Ten primers were found in the literature [29–32, 63, 64]. Primers Wspecf, Wspecr, *Wsp* 81F, *Wsp* 691 R, *ftsZ*f1 and *ftsZ*r1 are *Wolbachia*-specific. Primers pA (27F) and pH (1492 R) are general bacterial primers and EHR 16SD and EHR 16SR are specific to the Anaplasmataceae.

Additionally, four Anaplasmataceae-specific primers (i.e., 16S 567F, 16S 712F and 16S 712R and 16S 1401R) targeting the 16S gene were designed. The DNA sequences of the 16S rRNA of 26 Anaplasmataceae species (Table 3) were aligned in MEGAX: Molecular Evolutionary Genetics Analysis [65]. Regions of the gene that were similar among all sequences were used to design the primers using Primer3 4.1.0 [66, 67] (Tables 1 and 2).

**Table 3:** 16S ribosomal RNA sequences compared to design primers 16S 567F, 16S 712F, 16S 712R and 16S 1401R

Species	Strain	Host	NCBI accession number
*Ehrlichia chaffeensis*	Arkansas		NR_074500.2
*Ehrlichia ruminantium*	Welgevonden		NR_074513.2
*Ehrlichia minasensis*	UFMG-EV		NR_148800.1
*Ehrlichia muris subsp. eauclairensis*	Wisconsin_h		NR_157649.1
*Ehrlichia canis*	Oklahoma		NR_118741.1
*Anaplasma odocoilei*	UMUM76		NR_118489.1
*Anaplasma phagocytophilum*	Webster		NR_044762.1
*Neorickettsia risticii*	Illinois		NR_074389.1
*Neorickettsia sennetsu*	Miyayama		NR_074386.1
*Wolbachia*	wTak	*Drosophila takahashii*	DQ412082.2
*Wolbachia*	wAnga-Mali	*Anopheles gambiae*	MF944223.1
*Wolbachia*	L14_wolb99F	*Anopheles claviger*	KJ512995.1
*Wolbachia*	wRi	*Drosophila simulans*	DQ412085.1
*Wolbachia*		*Cacoxenus indagator*	EU930865.1
*Wolbachia*		*Diaphorina citri*	AB038370.1
*Wolbachia*		*Phloeomyzus passerinii*	JN109168.1
*Wolbachia*		*Mindarus japonicus*	JN109166.1
*Wolbachia*		*Hotaria unmunsana*	EU930866.1
*Wolbachia*		*Muscidifurax uniraptor*	L02882.1
*Wolbachia*	wAme	*Aphytis melinus*	EU981291.1
*Wolbachia*		*Trichogramma bourarachae*	AF062592.1
*Wolbachia*		*Osmia cornifrons*	EU930864.1
*Wolbachia*	A	*Mythimna separata*	EU753164.1
*Wolbachia*		*Onchocerca ochengi*	AF172401.1
*Wolbachia*		*Dirofilaria repens*	KY114937.1
*Wolbachia*	wIric 217F	*Ixodus ricinus*	EF219197.1

Two *Taq* polymerases were used; KAPA *Taq* polymerase (KAPA Biosystems, Cape Town, South Africa), using the manufacturers instruction and MyTaq *Taq* polymerase (Meridian Bioscience, Cincinnati, USA). The total reaction volume of 25.5 µL contained 18.25 µL of Sabax water, 5 µL of MyTaq reaction Buffer, 0.5 µL of each primer diluted to 10 µM, 0.25 µL of MyTaq *Taq* polymerase and 1 µL of DNA (≈ 100 ng). The MyTaq *Taq* polymerase has a higher specificity than the KAPA *Taq* polymerase. The KAPA *Taq* polymerase would often amplify products when MyTaq *Taq* polymerase did not. However, the KAPA *Taq* polymerase also led to multiple product amplifications. A total of four different cycling protocols (Additional file 11: Table S1) were tested. From the amplified products 2 µL were mixed with 1 µL of 30X Gelred (BIOTIUL, Hayward, California, USA) and visualized using agarose gel electrophoresis on a 2% agarose gel using BioRad Gel Doc™ Ez Imager and the software Image Lab 4.0.

### DNA sanger sequencing

Amplicons were characterised through DNA Sanger sequencing. The PCR amplicons were purified using 6% Sephadex G-50 gel filtration (Merck KGaA, Darmstadt, Germany). The purified products were visualized on an agarose gel using the protocol described above. For sequencing, we used a 10 µL sequencing reaction volume containing 5.5 µL of PCR grade water, 1 µL of BigDye™ (Applied BioSystems, Foster City, USA), 1 µL of sequencing buffer, 0.5 µL of primer diluted to 10 µM and 2 µL of purified PCR product. The cycling conditions included one cycle at 96 °C for 2 min, followed by 30 cycles of 30 s at 96 °C, 15 s at 50 °C and 4 min at 60 °C. Cycle sequencing products were purified using Sephadex G-50 gel filtration. Sequencing was performed on the ABI Prism™ 3500xl automated DNA sequencer (Applied Biosystems USA, Foster City, California, USA) at the University of Pretoria sequencing facility. The reverse and forward sequences obtained were aligned on CLC Main Workbench 8 (Qiagen, Hilden, Germany) and the consensus sequence was used for a BLASTn analysis [68] against the NCBI nucleotide database [69].

### Horizontal gene transfer from *Wolbachia* to *S. noctilio*

The *S. noctilio* genome assembly used in this study has been sequenced and assembled by Postma et al. (unpublished). Briefly, the *S. noctilio* genome was assembled and scaffolded into 6250 scaffolds using VelvetOptimiser [70] and SSPACE [71]. The genome assembly is estimated to be 185 Mb in size, with a N50 of 825 kb. The completeness of this genome assembly was estimated at 96.6% using BUSCO [72].

### Local BLAST using *Wolbachia* genomes against the *S. noctilio* genome

The first approach used to locate putative *Wolbachia* sequences in the genome of *S. noctilio* was series of local BLAST [68] searches, using complete *Wolbachia* genomes as queries against the genome of *S. noctilio*. The complete genomes of 14 *Wolbachia* strains were downloaded from NCBI [69] (Table 4). BLASTn analyses were performed using the 14 *Wolbachia* genomes as query and the *S. noctilio* genome as a reference sequence (0.001 e-value cutoff). The first BLASTn analysis only included eleven *Wolbachia* strains chosen either for the quality of their annotation or because their hosts belonged to the Hymenoptera family (i.e. wPip, wInc_Cu, wMel, wNo, GBW, wUni, wWitB, wNfla, wTpre) (Table 4).

**Table 4:** *Wolbachia* genomes used for a BLASTn analysis against the genome of *S. noctilio*

*Wolbachia* strain	Host	Assembly size	Number of scaffolds	GenBank accession
wCauA	*Carposina sasakii*	1,449,344	1	GCA_006542295.1
wCfeJ	*Ctenocephalides felis*	1,201,647	1	GCA_012277315.1
wPip	*Culex quinquefasciatus*	1,482,455	1	GCA_000073005.1
wDi	*Diaphorina citri*	1,656,288	1	GCA_013458815.1
wAna	*Drosophila ananassae*	1,401,460	1	GCA_008033215.1
wInc_Cu	*Drosophila incompta*	1,267,840	1	GCA_001758565.1
wMel	*Drosophila melanogaster*	1,267,782	1	GCA_000008025.1
wNo	*Drosophila simulans*	1,301,823	1	GCA_000376585.1
	*Formica exsecta*	3,096,460	69	GCA_003704235.1
GBW	*Leptopilina clavipes*	1,150,755	46 (contigs)	GCA_006334525.1
wUni	*Muscidifurax uniraptor*	867,873	256	GCA_000174095.1
wWitB	*Nasonia vitripennis*	1,107,643	426	GCA_000204545.1
wNfla	*Nomada flava*	1,332,780	167 (contigs)	GCA_001675695.1
wTpre	*Trichogramma pretiosum*	1,133,809	1	GCA_001439985.1

Subsequently, the genomic sequences from *S. noctilio* which exhibited significant similarity to *Wolbachia* were subjected to BLASTx analyses [68] against the NCBI protein database [69]. These sequences helped to identify four additional *Wolbachia* strains (i.e., wCauA, wCfeJ, wDi, wAna) (Table 4) with a higher percent identity than the previously identified eleven strains. We then added the complete genomes of these four strains to that of the previous eleven and executed a second BLASTn analysis.

### Taxonomic classification of *S. noctilio* sequence data

The second approach used to identify *Wolbachia* sequences in the *S. noctilio* genome was a taxonomic classification of genomic DNA reads from *S. noctilio* using Kraken 2 [73]. The DNA reads were compared to the standard Kraken2 database.

### Whole genome alignment using MUMmer

The third approach used to identify *Wolbachia* sequences in the *S. noctilio* genome was a series of whole genome alignments using MUMmer [74]. The genome of *S. noctilio* was aligned to the genomes of four *Wolbachia* strains (i.e., wCauA, wCfeJ, wDi, wInc_Cu) (**Table ****4**).

### BLAST of scaffolds from the *S. noctilio* genome against NCBI

The BLASTn [57] analysis and the taxonomic classification methods both identified scaffolds within the *S. noctilio* genome assembly that potentially contained *Wolbachia* sequences. To determine the position and length of these sequences as well as identify possible *Wolbachia* genes on the identified scaffolds, we used the full scaffolds for a BLASTx analysis [68] against the NCBI protein database [69]. This also allowed us to extract the DNA sequences of the horizontally transferred genes and to annotate them.

### Phylogenetic relationships of candidate horizontally transferred *Wolbachia* genes

To confirm that the genes identified were transferred from *Wolbachia* and were not of eukaryotic origin, we constructed individual gene phylogenies. A BLASTx analysis [68] was performed against the protein database of NCBI [69]. The output of the BLASTx analysis was filtered by selecting sequences extracted from fully sequenced *Wolbachia* genomes. Whenever possible, the protein sequences used as outgroups were selected from bacterial species belonging to taxa outside of the alphaproteobacteria. However, for ORF1, ORF10, ORF11 and ORF12, similar sequences could only be found in other *Wolbachia* strains or in other Rickettsiales.

Each dataset was aligned in MEGA X: Molecular Evolutionary Genetics Analysis [65] using the Clustal W alignment tool and the default parameters. The sequences were then trimmed manually and the reference sequences that did not overlap with the sequences from the *S. noctilio* genome were taken out. A maximum likelihood analysis was performed in IQ-TREE 2 [75] using 1000 bootstrap replicates. The best substitution models were selected using ModelFinder [76]. The phylogenetic trees were edited in iTOL [77].

### Ubiquity of horizontally transferred genes in *S. noctilio* in South Africa

Once the sequences of the horizontally transferred genes were identified, we used these sequences to design six primers using Primer3 4.0.1 [66, 67] (Tables 1 and 2). These primers allowed us to screen for the presence of the horizontally transferred *Wolbachia* genes in various populations of *S. noctilio* in South Africa, and to confirm that those genes are ubiquitous in these populations. We sampled 100 individuals from five populations that correspond to five pine growing regions in South Africa; Western Cape, Southern Cape, Eastern Cape, KwaZulu-Natal and Mpumalanga. The sampling process was similar to previously described except for the fact that only males were sampled for this experiment. After dissection, the DNA was extracted using the *prep*GEM Insect DNA extraction kit (ZyGEM Corporation Ltd, Hamilton, New Zealand) and the PCR amplification was done using the KAPA *Taq* PCR kit (KAPA Biosystems, Cape Town South Africa) as previously described. The DNA purification process, visualization of the PCR amplicons and sequencing protocol are as described above.

To confirm that primers SnW1f and SnW1r, SnW2f and SnW2r and SnW3f and SnW3r were amplifying the desired *Wolbachia* sequences, the PCR amplicons from one female and from one male sample for each of the six different primers were sequenced. The sequences obtained were used for a BLASTn analysis [68] against the *S. noctilio* genome in CLC Main Workbench 8 (Qiagen, Hilden, Germany). Those samples were used as positive controls for the remaining PCRs. When visualizing the PCR amplicons using agarose gel electrophoresis, the presence of a band at the same height as the positive control indicated the presence of the horizontally transferred *Wolbachia* gene in the sampled individual. The quantity of DNA in the samples showing no bands was measured using a nanodrop and the DNA was then diluted to obtain a DNA concentration around 100 ng/nL.

## Supplementary Information

**Additional file 1: Figure S1. **Maximum likelihood tree. It was constructed with the protein sequence of ORF1 compared to similar protein sequences of 11 *Wolbachia* strains and one protein sequence from *Diplorickettsia massiliensis* (Gammaproteobacteria: Coxiellaceae) (out group). The branch indicated in red represents the position of ORF1 among other *Wolbachia* protein sequences. All *Wolbachia* strains are named after their hosts as follows: wAus, *Plutella australiana*; wCauA, *Carposina sasakii*; wDi, *Diaphorina citri*; wNfla, *Nomada flava*; wNleu, *Nomada leucophthalma*; wNo, *Drosophila simulans*; wNpa, *Nomada panzeri*; wPip, *Culex quinquefasciatus; *wPnig, *Pentalonia nigronervosa*; wStri, *Laodelphax striatellus*; wVulC, *Armadillidium vulgare*.

**Additional file 2: Figure S2. **Maximum likelihood tree. It was constructed with the protein sequence of ORF2 compared to similar protein sequences of 12 *Wolbachia* strains and one protein sequence from *Herpetosiphon llansteffanense* (Terrabacteria: Herpetosiphonales) (out group). The branch indicated in red represents the position of ORF2 among other *Wolbachia* protein sequences. All *Wolbachia* strains are named after their hosts as follows: wAna, *Drosophila ananassae*; wCauA, *Carposina sasakii*; wCobs, *Cardiocondyla obscurior*; wCon, *Cylisticus convexus*; wHa, *Drosophila simulans*; wKgib, *Kradibia gibbosae*; wLug, *Nilaparvata lugens*; wMelPop, *Drosophila melanogaster*; wPnig, *Pentalonia nigronervosa*; wUni, *Muscidifurax uniraptor*; wTpre, *Trichogramma pretiosum; *wVulC, *Armadillidium vulgare*.

**Additional file 3: Figure S3. **Maximum likelihood tree. It was constructed with the protein sequence of ORF3 compared to similar protein sequences of two *Wolbachia* strains and one protein sequence from *Mastigocladopsis repens* (Cyanobacteria: Symphyonemataceae) (out group). The branch indicated in red represents the position of ORF3 among other protein sequences. The two *Wolbachia* strains are named after their hosts as follows: wFcan, *Folsomia candida*; wVulC, *Armadillidium vulgare*.

**Additional file 4: Figure S4. **Maximum likelihood tree. It was constructed with the protein sequence of ORF4 compared to similar protein sequences of seven *Wolbachia* strains and one protein sequence from *Legionella pneumophila* (Gammaproteobacteria: Legionellaceae) (out group). The branch indicated in red represents the position of ORF4 among other *Wolbachia* protein sequences. All *Wolbachia* strains are named after their hosts as follows: wAu, *Drosophila simulans*; wDac, *Dactylopius coccus*; wHa, *Drosophila simulans*; wMelPop, *Drosophila melanogaster*; wOne, *Nasonia oneida*; wUni, *Muscidifurax uniraptor*; wVulC, *Armadillidium vulgare*.

**Additional file 5: Figure S5. **Maximum likelihood tree. It was constructed with the protein sequence of ORF6 compared to similar protein sequences of 23 *Wolbachia* strains and one protein sequence from *Holospora undulata* (Alphaproteobacteria: Holosporaceae) (out group). The branch indicated in red represents the position of ORF6 among other *Wolbachia* protein sequences. All *Wolbachia* strains are named after their hosts as follows: wAna, *Drosophila ananassae*; wBt, *Bemisia tabaci*; wCauA, *Carposina sasakii*; wCobs, *Cardiocondyla obscurior*; wCon, *Cylisticus convexus*; wDac, *Dactylopius coccus*; wDi, *Diaphorina citri*; wFcan, *Folsomia candida*; wKgib, *Kradibia gibbosae*; wLug, *Nilaparvata lugens*; wMau, *Drosophila mauritiana*; wMeg, *Chrysomya megacephala*; wMelPop, *Drosophila melanogaster*; wNfe, *Nomada ferruginata*; wNo, *Drosophila simulans*; wOne, *Nasonia oneida*; wPip, *Culex quinquefasciatus*; wPip_Mol, *Culex molestus*; wPnig, *Pentalonia nigronervosa*; wStri, *Laodelphax striatellus*; wTei, *Drosophila teissieri*; wVulC, *Armadillidium vulgare*; wYak, *Drosophila yakuba*.

**Additional file 6: Figure S6. **Maximum likelihood tree. It was constructed with the protein sequence of ORF7 compared to similar protein sequences of 22 *Wolbachia* strains and one protein sequence from *Holospora undulata* (Alphaproteobacteria: Holosporaceae). The branch indicated in red represents the position of ORF7 among other *Wolbachia* protein sequences. All *Wolbachia* strains are named after their hosts as follows: wBt, *Bemisia tabaci*; wCauA, *Carposina sasakii*; wCfeT, *Ctenocephalides felis*; wCobs, *Cardiocondyla obscurior*; wCon, *Cylisticus convexus*; wDac, *Dactylopius coccus*; wDi, *Diaphorina citri*; wFcan, *Folsomia candida*; wGmo, *Glossina morsitans*; wInc, *Drosophila incompta*; wKgib, *Kradibia gibbosae*; wLug, *Nilaparvata lugens*; wMau, *Drosophila mauritiana*; wMeg, *Chrysomya megacephala*; wNleu, *Nomada leucophthalma*; wNo, *Drosophila simulans*; wNpa, *Nomada panzeri*; wPip, *Culex quinquefasciatus*; wPnig, *Pentalonia nigronervosa*; wStri, *Laodelphax striatellus*; wVulC, *Armadillidium vulgare*.

**Additional file 7: Figure S7. **Maximum likelihood tree. It was constructed with the protein sequence of ORF9 compared to similar protein sequences of 20 *Wolbachia* strains and one protein sequence from *Moorea producens* (Cyanobacteria: Oscillatoriaceae). The branch indicated in red represents the position of ORF9 among other *Wolbachia* protein sequences. All *Wolbachia* strains are named after their hosts as follows: wAna, *Drosophila ananassae*; wAu, *Drosophila simulans*; wBt, *Bemisia tabaci*; wCfeT, *Ctenocephalides felis*; wCon, *Cylisticus convexus*; wDac, *Dactylopius coccus*; wDi, *Diaphorina citri*; wInc, *Drosophila incompta*; wKgib, *Kradibia gibbosae*; wMeg, *Chrysomya megacephala*; wMel, *Drosophila melanogaster*; wOb, *Operophtera brumata*; wOne, *Nasonia oneida*; wPip, *Culex quinquefasciatus*; wPol, *Atemnus politus*; wSan, *Drosophila santomea*; wStri, *Laodelphax striatellus*; wTei, *Drosophila teissieri*; wVulC, *Armadillidium vulgare*; wYak, *Drosophila yakuba*.

**Additional file 8: Figure S8. **Maximum likelihood tree. It was constructed with the protein sequence of ORF10 compared to similar protein sequences of 21 *Wolbachia* strains and one protein sequence from *Diplorickettsia massiliensis* (Gammaproteobacteria: Coxiellaceae). The branch indicated in red represents the position of ORF10 among other *Wolbachia* protein sequences. All *Wolbachia* strains are named after their hosts as follows: wAlbB, *Aedes albopictus* ; wAna, *Drosophila ananassae*; wAus, *Plutella australiana* ; wCauA, *Carposina sasakii*; wCfeJ, *Ctenocephalides felis*; wCle, *Cimex lectularius*; wCobs, *Cardiocondyla obscurior*; wCon, *Cylisticus convexus*; wDi, *Diaphorina citri*; wFcan, *Folsomia candida*; wMau, *Drosophila mauritiana*; wMel, *Drosophila melanogaster*; wNfe, *Nomada ferruginata*; wNo, *Drosophila simulans*; wOb, *Operophtera brumata*; wPip, *Culex quinquefasciatus*; wPnig, *Pentalonia nigronervosa*; wSan, *Drosophila santomea*; wStri, *Laodelphax striatellus*; wVulC, *Armadillidium vulgare.*

**Additional file 9: Figure S9. **Maximum likelihood tree. It was constructed with the protein sequence of ORF11 compared to similar protein sequences of 10 *Wolbachia* strains. The branch indicated in red represents the position of ORF11 among other *Wolbachia* protein sequences. All *Wolbachia* strains are named after their hosts as follows: wAlbB, *Aedes albopictus*; wAus, *Plutella australiana*; wBlon, *Brontispa longissima*; wCobs, *Cardiocondyla obscurior*; wDi, *Diaphorina citri*; wMau, *Drosophila mauritiana*; wNo, *Drosophila simulans*; wPip, *Culex quinquefasciatus*; wPnig, *Pentalonia nigronervosa*; wStri, *Laodelphax striatellus*.

**Additional file 10: Figure S10. **Maximum likelihood tree. It was constructed with the protein sequence of ORF12 compared to similar protein sequences of seven *Wolbachia* strains. The branch indicated in red represents the position of ORF12 among other *Wolbachia* protein sequences. All *Wolbachia* strains are named after their hosts as follows: wAlbB, *Aedes albopictus*; wAus, *Plutella australiana*; wDi, *Diaphorina citri*; wPip, *Culex quinquefasciatus*; wPip_Mol, *Culex molestus*; wPnig, *Pentalonia nigronervosa*; wStri, *Laodelphax striatellus*.

**Additional file 11: Table S1. **PCR cycling protocol. Tm = Annealing temperature specific to the primer pair (Table 2); * T° decreases by 0.5°C at the start of each cycle.

## Data Availability

The sequences of the amplicons obtained using the primers designed in this study are available in GenBank under the following accession numbers: SnW1f and SnW1r, MW848339; SnW2f and SnW2r, MW848340; SnW3f and SnW3r, MW848341.

## Abbreviations

HGT: Horizontal gene transfer
ORF: Open reading frame
