# Supplementary figures and images for: Remnants of horizontal transfers of Wolbachia genes in a Wolbachia-free woodwasp
Source: BMC Ecol Evol. 2022 Mar 26;22:36. doi: 10.1186/s12862-022-01995-x (PMC8962096; doi:10.1186/s12862-022-01995-x)

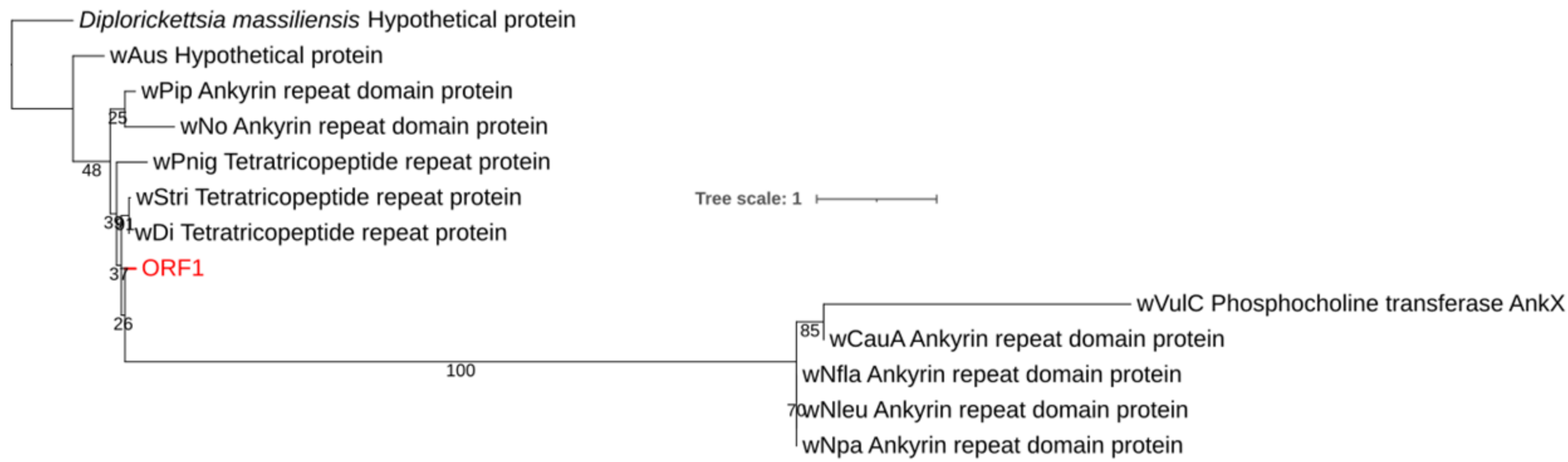

Supplement: Supplementary file 1 — Additional file 1: Figure S1. Maximum likelihood tree. It was constructed with the protein sequence of ORF1 compared to similar protein sequences of 11 Wolbachia strains and one protein sequence from Diplorickettsia massiliensis (Gammaproteobacteria: Coxiellaceae) (out group). The branch indicated in red represents the position of ORF1 among other Wolbachia protein sequences. All Wolbachia strains are named after their hosts as follows: wAus, Plutella australiana; wCauA, Carposina sasakii; wDi, Diaphorina citri; wNfla, Nomada flava; wNleu, Nomada leucophthalma; wNo, Drosophila simulans; wNpa, Nomada panzeri; wPip, Culex quinquefasciatus; wPnig, Pentalonia nigronervosa; wStri, Laodelphax striatellus; wVulC, Armadillidium vulgare. [file 12862_2022_1995_MOESM1_ESM.pdf]

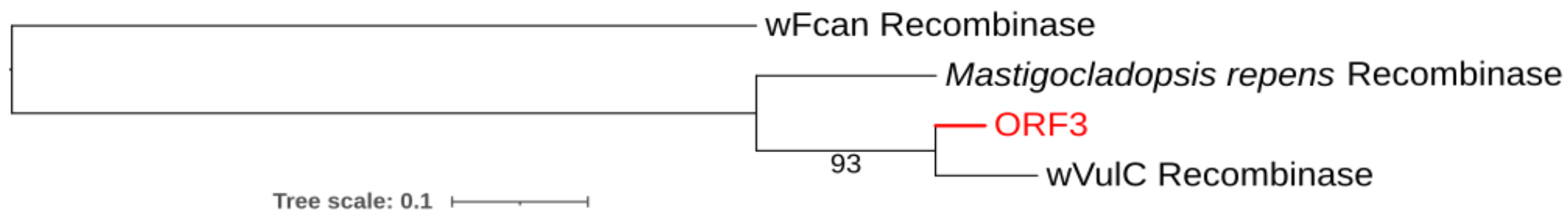

Supplement: Supplementary file 3 — Additional file 3: Figure S3. Maximum likelihood tree. It was constructed with the protein sequence of ORF3 compared to similar protein sequences of two Wolbachia strains and one protein sequence from Mastigocladopsis repens (Cyanobacteria: Symphyonemataceae) (out group). The branch indicated in red represents the position of ORF3 among other protein sequences. The two Wolbachia strains are named after their hosts as follows: wFcan, Folsomia candida; wVulC, Armadillidium vulgare. [file 12862_2022_1995_MOESM3_ESM.pdf]

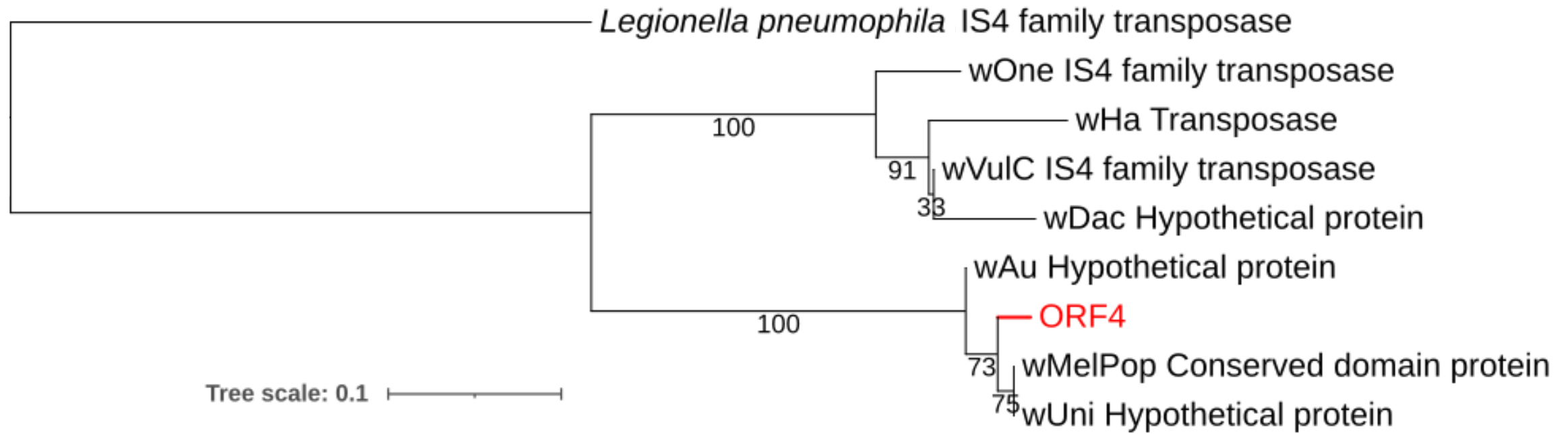

Supplement: Supplementary file 4 — Additional file 4: Figure S4. Maximum likelihood tree. It was constructed with the protein sequence of ORF4 compared to similar protein sequences of seven Wolbachia strains and one protein sequence from Legionella pneumophila (Gammaproteobacteria: Legionellaceae) (out group). The branch indicated in red represents the position of ORF4 among other Wolbachia protein sequences. All Wolbachia strains are named after their hosts as follows: wAu, Drosophila simulans; wDac, Dactylopius coccus; wHa, Drosophila simulans; wMelPop, Drosophila melanogaster; wOne, Nasonia oneida; wUni, Muscidifurax uniraptor; wVulC, Armadillidium vulgare. [file 12862_2022_1995_MOESM4_ESM.pdf]

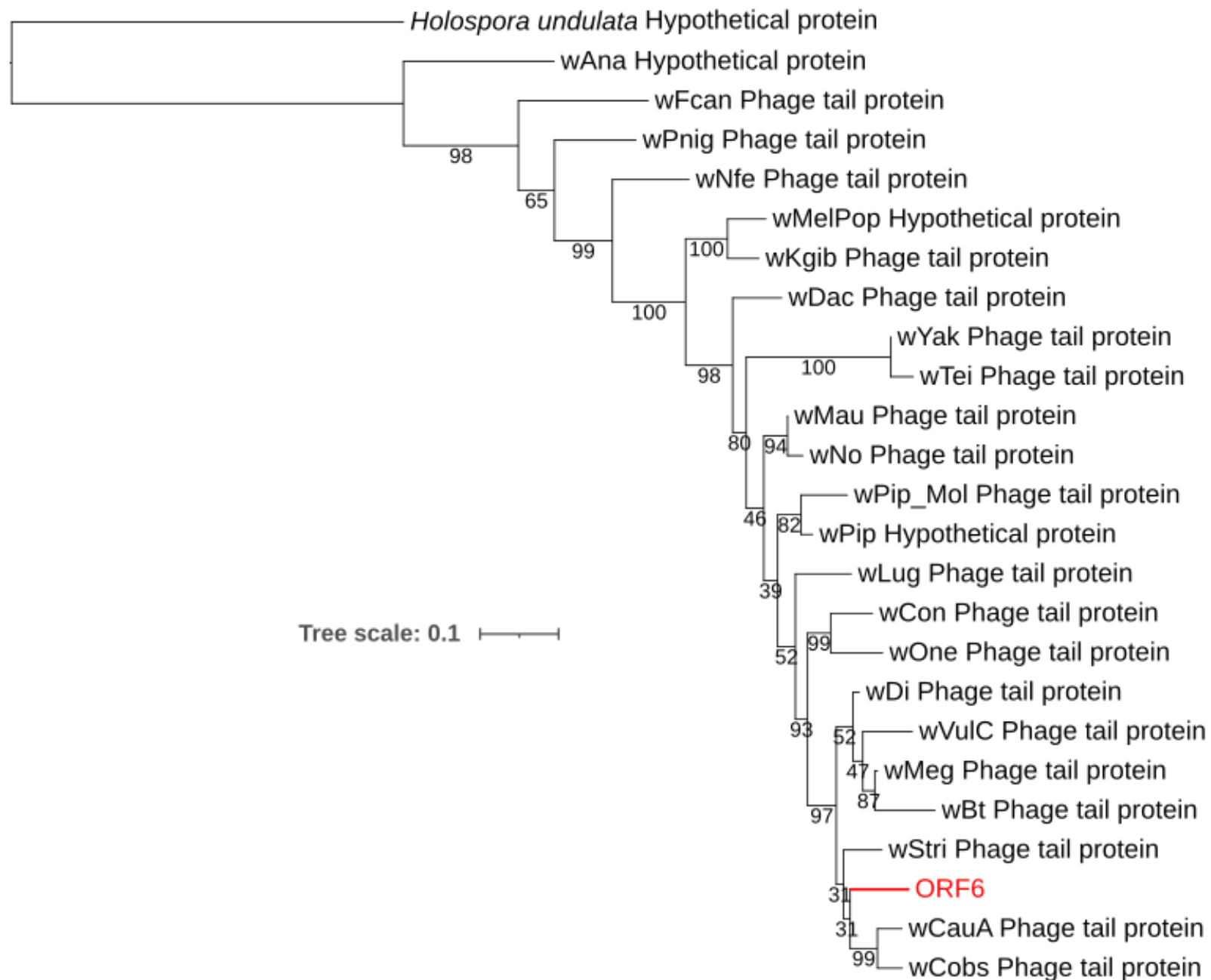

Supplement: Supplementary file 5 — Additional file 5: Figure S5. Maximum likelihood tree. It was constructed with the protein sequence of ORF6 compared to similar protein sequences of 23 Wolbachia strains and one protein sequence from Holospora undulata (Alphaproteobacteria: Holosporaceae) (out group). The branch indicated in red represents the position of ORF6 among other Wolbachia protein sequences. All Wolbachia strains are named after their hosts as follows: wAna, Drosophila ananassae; wBt, Bemisia tabaci; wCauA, Carposina sasakii; wCobs, Cardiocondyla obscurior; wCon, Cylisticus convexus; wDac, Dactylopius coccus; wDi, Diaphorina citri; wFcan, Folsomia candida; wKgib, Kradibia gibbosae; wLug, Nilaparvata lugens; wMau, Drosophila mauritiana; wMeg, Chrysomya megacephala; wMelPop, Drosophila melanogaster; wNfe, Nomada ferruginata; wNo, Drosophila simulans; wOne, Nasonia oneida; wPip, Culex quinquefasciatus; wPip_Mol, Culex molestus; wPnig, Pentalonia nigronervosa; wStri, Laodelphax striatellus; wTei, Drosophila teissieri; wVulC, Armadillidium vulgare; wYak, Drosophila yakuba. [file 12862_2022_1995_MOESM5_ESM.pdf]

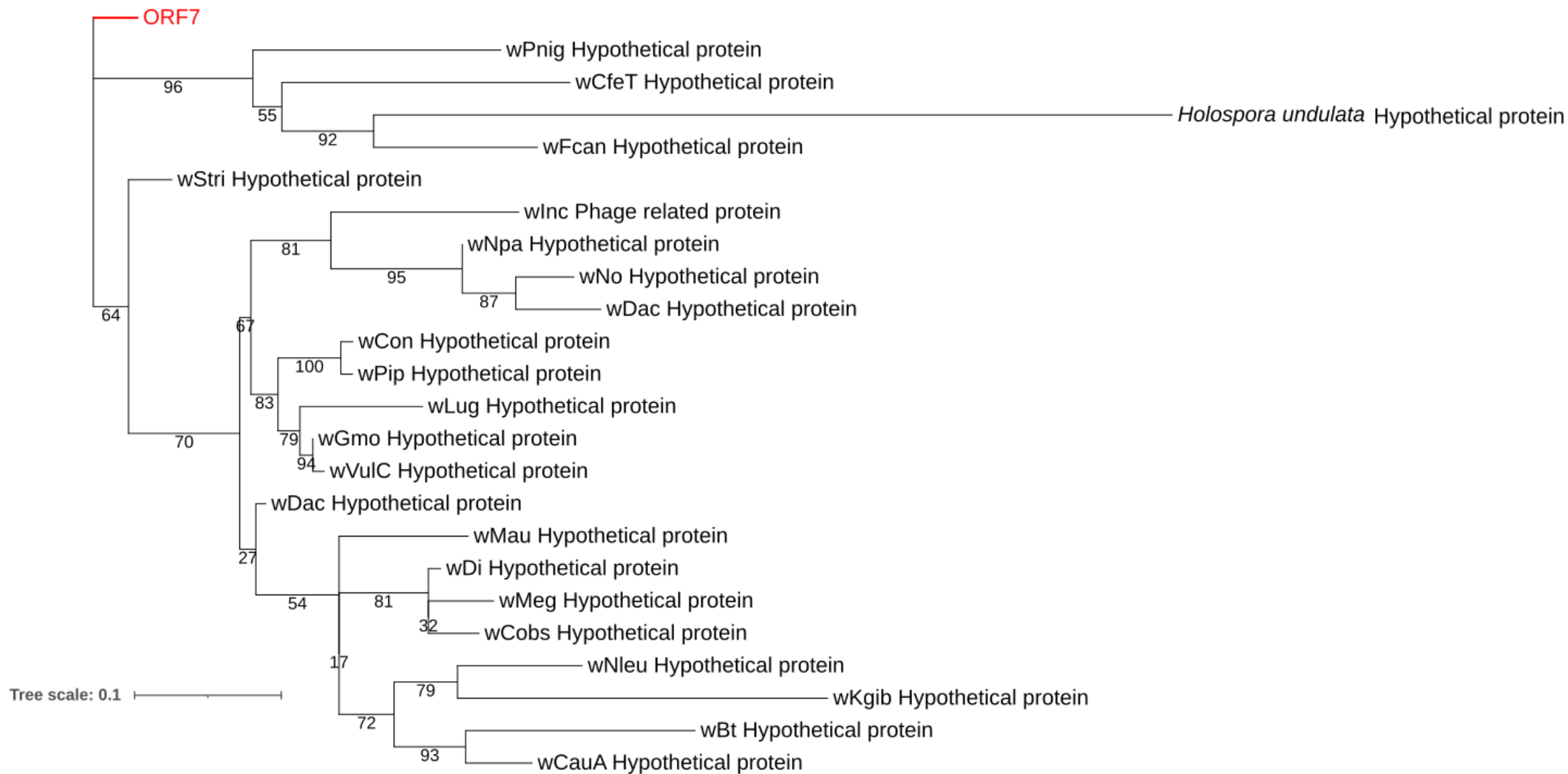

Supplement: Supplementary file 6 — Additional file 6: Figure S6. Maximum likelihood tree. It was constructed with the protein sequence of ORF7 compared to similar protein sequences of 22 Wolbachia strains and one protein sequence from Holospora undulata (Alphaproteobacteria: Holosporaceae). The branch indicated in red represents the position of ORF7 among other Wolbachia protein sequences. All Wolbachia strains are named after their hosts as follows: wBt, Bemisia tabaci; wCauA, Carposina sasakii; wCfeT, Ctenocephalides felis; wCobs, Cardiocondyla obscurior; wCon, Cylisticus convexus; wDac, Dactylopius coccus; wDi, Diaphorina citri; wFcan, Folsomia candida; wGmo, Glossina morsitans; wInc, Drosophila incompta; wKgib, Kradibia gibbosae; wLug, Nilaparvata lugens; wMau, Drosophila mauritiana; wMeg, Chrysomya megacephala; wNleu, Nomada leucophthalma; wNo, Drosophila simulans; wNpa, Nomada panzeri; wPip, Culex quinquefasciatus; wPnig, Pentalonia nigronervosa; wStri, Laodelphax striatellus; wVulC, Armadillidium vulgare. [file 12862_2022_1995_MOESM6_ESM.pdf]

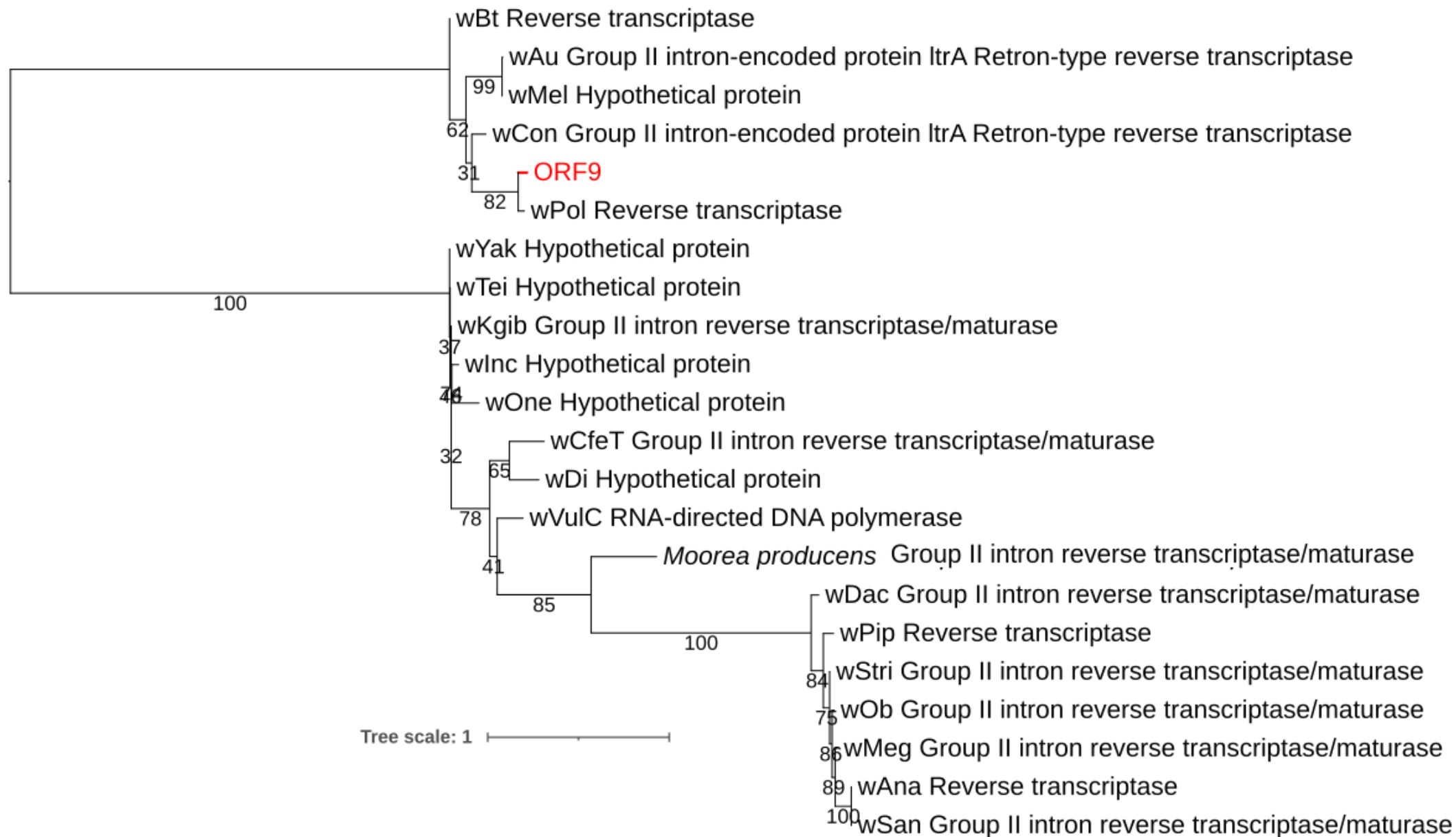

Supplement: Supplementary file 7 — Additional file 7: Figure S7. Maximum likelihood tree. It was constructed with the protein sequence of ORF9 compared to similar protein sequences of 20 Wolbachia strains and one protein sequence from Moorea producens (Cyanobacteria: Oscillatoriaceae). The branch indicated in red represents the position of ORF9 among other Wolbachia protein sequences. All Wolbachia strains are named after their hosts as follows: wAna, Drosophila ananassae; wAu, Drosophila simulans; wBt, Bemisia tabaci; wCfeT, Ctenocephalides felis; wCon, Cylisticus convexus; wDac, Dactylopius coccus; wDi, Diaphorina citri; wInc, Drosophila incompta; wKgib, Kradibia gibbosae; wMeg, Chrysomya megacephala; wMel, Drosophila melanogaster; wOb, Operophtera brumata; wOne, Nasonia oneida; wPip, Culex quinquefasciatus; wPol, Atemnus politus; wSan, Drosophila santomea; wStri, Laodelphax striatellus; wTei, Drosophila teissieri; wVulC, Armadillidium vulgare; wYak, Drosophila yakuba. [file 12862_2022_1995_MOESM7_ESM.pdf]

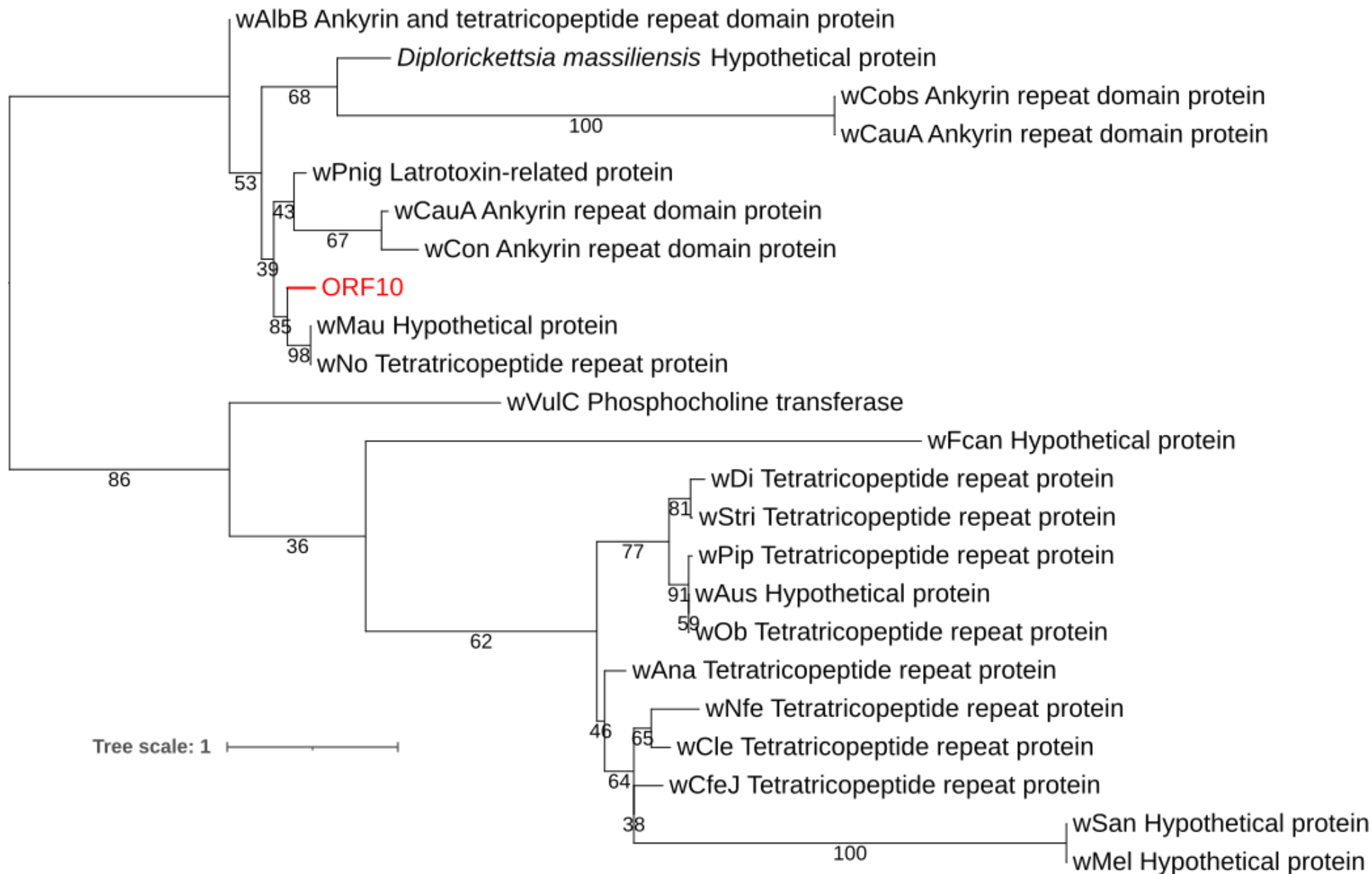

Supplement: Supplementary file 8 — Additional file 8: Figure S8. Maximum likelihood tree. It was constructed with the protein sequence of ORF10 compared to similar protein sequences of 21 Wolbachia strains and one protein sequence from Diplorickettsia massiliensis (Gammaproteobacteria: Coxiellaceae). The branch indicated in red represents the position of ORF10 among other Wolbachia protein sequences. All Wolbachia strains are named after their hosts as follows: wAlbB, Aedes albopictus ; wAna, Drosophila ananassae; wAus, Plutella australiana ; wCauA, Carposina sasakii; wCfeJ, Ctenocephalides felis; wCle, Cimex lectularius; wCobs, Cardiocondyla obscurior; wCon, Cylisticus convexus; wDi, Diaphorina citri; wFcan, Folsomia candida; wMau, Drosophila mauritiana; wMel, Drosophila melanogaster; wNfe, Nomada ferruginata; wNo, Drosophila simulans; wOb, Operophtera brumata; wPip, Culex quinquefasciatus; wPnig, Pentalonia nigronervosa; wSan, Drosophila santomea; wStri, Laodelphax striatellus; wVulC, Armadillidium vulgare. [file 12862_2022_1995_MOESM8_ESM.pdf]

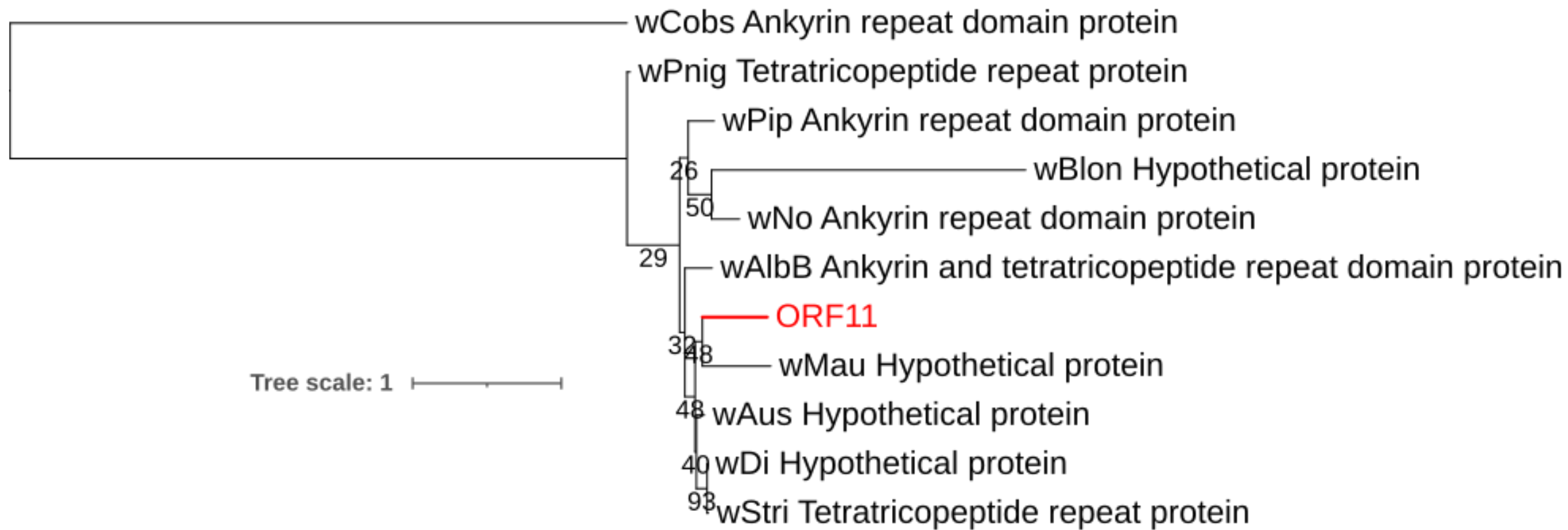

Supplement: Supplementary file 9 — Additional file 9: Figure S9. Maximum likelihood tree. It was constructed with the protein sequence of ORF11 compared to similar protein sequences of 10 Wolbachia strains. The branch indicated in red represents the position of ORF11 among other Wolbachia protein sequences. All Wolbachia strains are named after their hosts as follows: wAlbB, Aedes albopictus; wAus, Plutella australiana; wBlon, Brontispa longissima; wCobs, Cardiocondyla obscurior; wDi, Diaphorina citri; wMau, Drosophila mauritiana; wNo, Drosophila simulans; wPip, Culex quinquefasciatus; wPnig, Pentalonia nigronervosa; wStri, Laodelphax striatellus. [file 12862_2022_1995_MOESM9_ESM.pdf]

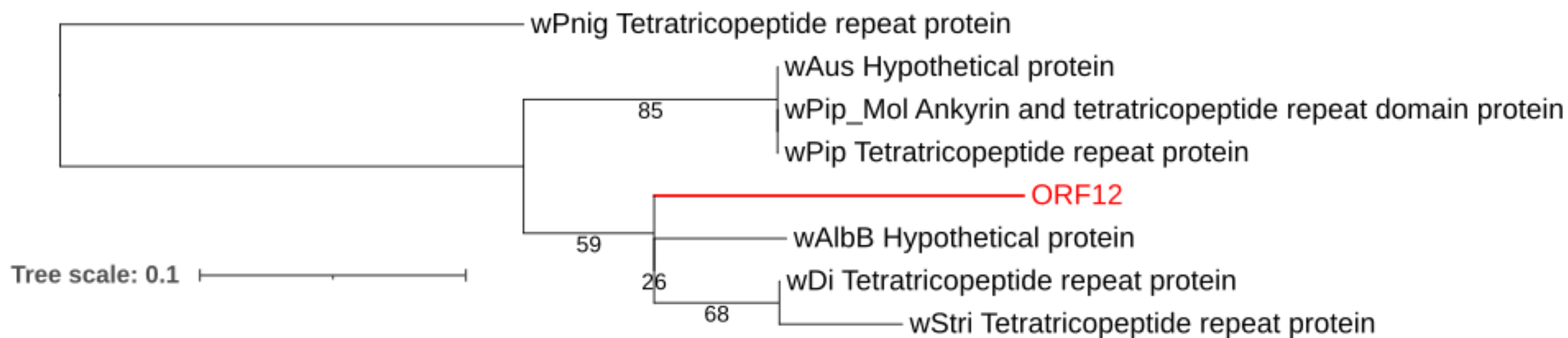

Supplement: Supplementary file 10 — Additional file 10: Figure S10. Maximum likelihood tree. It was constructed with the protein sequence of ORF12 compared to similar protein sequences of seven Wolbachia strains. The branch indicated in red represents the position of ORF12 among other Wolbachia protein sequences. All Wolbachia strains are named after their hosts as follows: wAlbB, Aedes albopictus; wAus, Plutella australiana; wDi, Diaphorina citri; wPip, Culex quinquefasciatus; wPip_Mol, Culex molestus; wPnig, Pentalonia nigronervosa; wStri, Laodelphax striatellus. [file 12862_2022_1995_MOESM10_ESM.pdf]
