# Supplementary material for: Remnants of horizontal transfers of Wolbachia genes in a Wolbachia-free woodwasp
Source: BMC Ecol Evol. 2022 Mar 26;22:36. doi: 10.1186/s12862-022-01995-x (PMC8962096; doi:10.1186/s12862-022-01995-x)
Supplement: Supplementary file 2 — Additional file 2: Figure S2. Maximum likelihood tree. It was constructed with the protein sequence of ORF2 compared to similar protein sequences of 12 Wolbachia strains and one protein sequence from Herpetosiphon llansteffanense (Terrabacteria: Herpetosiphonales) (out group). The branch indicated in red represents the position of ORF2 among other Wolbachia protein sequences. All Wolbachia strains are named after their hosts as follows: wAna, Drosophila ananassae; wCauA, Carposina sasakii; wCobs, Cardiocondyla obscurior; wCon, Cylisticus convexus; wHa, Drosophila simulans; wKgib, Kradibia gibbosae; wLug, Nilaparvata lugens; wMelPop, Drosophila melanogaster; wPnig, Pentalonia nigronervosa; wUni, Muscidifurax uniraptor; wTpre, Trichogramma pretiosum; wVulC, Armadillidium vulgare. [file 12862_2022_1995_MOESM2_ESM.pdf]

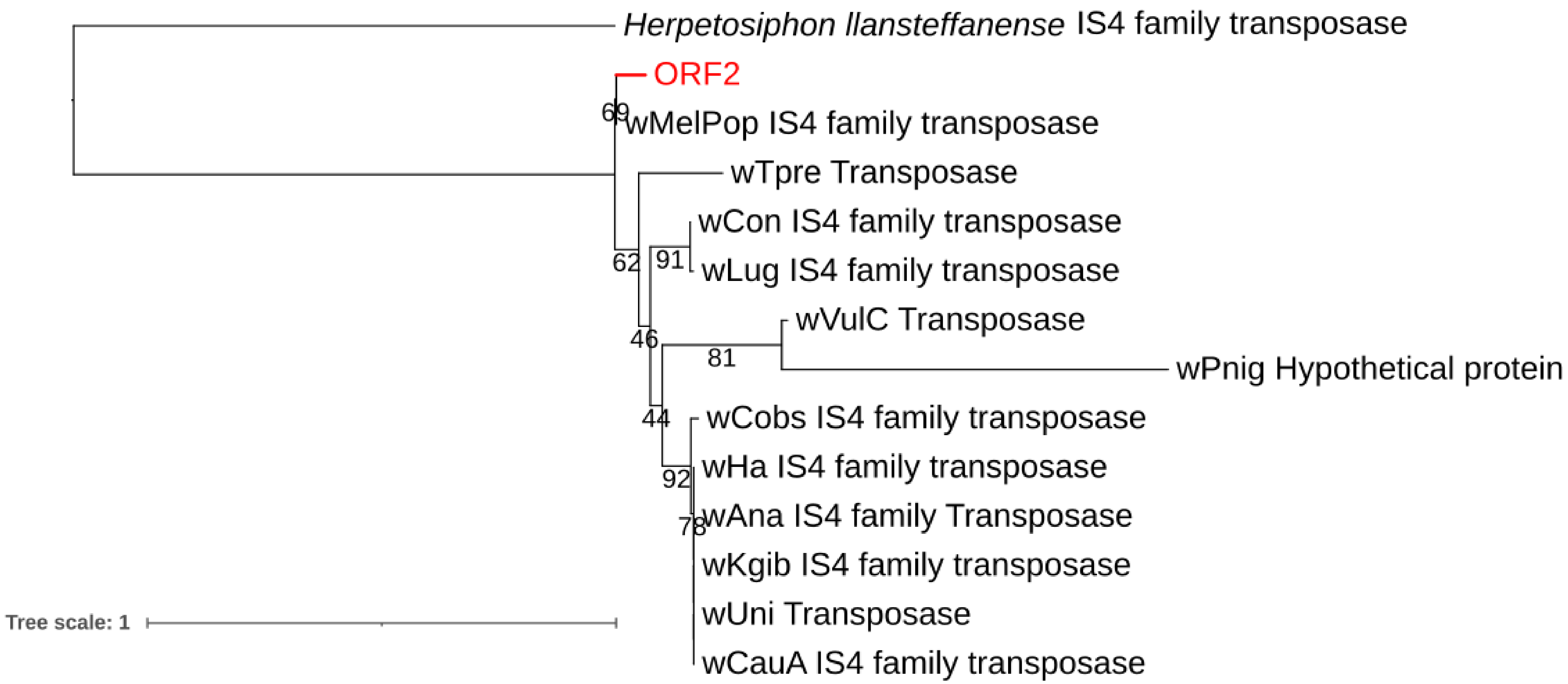

Herpetosiphon llansteffanense IS4 family transposase
ORF2
wMelPop IS4 family transposase
wTpre Transposase
wCon IS4 family transposase
wLug IS4 family transposase
wVulC Transposase
wPnig Hypothetical protein
wCobs IS4 family transposase
wHa IS4 family transposase
wAna IS4 family Transposase
wKgib IS4 family transposase
wUni Transposase
wCauA IS4 family transposase
69
62
91
46
81
44
92
78
Tree scale: 1
