# Supplementary material for: Remnants of horizontal transfers of Wolbachia genes in a Wolbachia-free woodwasp
Source: BMC Ecol Evol. 2022 Mar 26;22:36. doi: 10.1186/s12862-022-01995-x (PMC8962096; doi:10.1186/s12862-022-01995-x)
Supplement: Supplementary file 11 — Additional file 11: Table S1. PCR cycling protocol. Tm = Annealing temperature specific to the primer pair (Table 2); * T° decreases by 0.5°C at the start of each cycle. [file 12862_2022_1995_MOESM11_ESM.docx]

| **Additional table 1: PCR cycling protocols.** | | | |
| --- | --- | --- | --- |
| Protocol | Number of cycles | Time | Temperature (°C) |
| Kapa Taq | 1 | 3 min | 95 |
|  | 35 | 30 sec | 95 |
|  |  | 30 sec | Tm |
|  |  | 1 min | 72 |
| MyTaq | 1 | 10 min | 94 |
|  | 30 | 1 min | 94 |
|  |  | 1 min | Tm |
|  |  | 1 min | 72 |
|  | 1 | 5 min | 72 |
| Touch down | 1 | 5 min | 94 |
|  | 20 | 30 sec | 94 |
|  |  | 45 sec | Tm + 10 * |
|  |  | 2 min | 72 |
|  | 20 | 30 sec | 94 |
|  |  | 45 sec | Tm |
|  |  | 2 min | 72 |
|  | 1 | 5 min | 72 |
| Trouble shooting | 1 | 5 min | 95 |
|  | 3 | 30 sec | 95 |
|  |  | 30 sec | 45 |
|  |  | 80 sec | 72 |
|  | 35 | 15 sec | 94 |
|  |  | 15 sec | Tm |
|  |  | 45 sec | 72 |
|  | 1 | 3 min | 72 |
| Tm = Annealing temperature specific to the primer pair (**Table 2**); * T° decreases by 0.5°C at the start of each cycle | | | |
